# Magnetically guided theranostics: montmorillonite-based iron/platinum nanoparticles for enhancing in situ MRI contrast and hepatocellular carcinoma treatment

**DOI:** 10.1186/s12951-021-01052-7

**Published:** 2021-10-09

**Authors:** Ming-Hsien Chan, Chih-Ning Lu, Yi-Lung Chung, Yu-Chan Chang, Chien-Hsiu Li, Chi-Long Chen, Da-Hua Wei, Michael Hsiao

**Affiliations:** 1 Genomics Research Center, Academia Sinica, Taipei, 115 Taiwan; 2 Department of Chemistry, Saint Michael’s College, Colchester, VT 05439 USA; 3 Graduate Institute of Manufacturing Technology and Department of Mechanical Engineering, National Taipei University of Technology, National Taipei University of Technology, Taipei, 106 Taiwan; 4 Department of Biomedical Imaging and Radiological Sciences, National Yang Ming Chiao Tung University, Taipei, 112 Taiwan; 5 Department of Pathology, College of Medicine, Department of Pathology, Taipei Medical University, Taipei Medical University Hospital, Taipei, 110 Taiwan; 6 Department of Biochemistry, College of Medicine, Kaohsiung Medical University, Kaohsiung, 807 Taiwan

**Keywords:** T2-weighted magnetic resonance imaging, Superparamagnetic FePt nanoparticles, Hepatocellular carcinoma, Drug delivery system, Nanocomposites

## Abstract

In Asia, including Taiwan, malignant tumors such as Hepatocellular carcinoma (HCC) one of the liver cancer is the most diagnosed subtype. Magnetic resonance imaging (MRI) has been a typical diagnostic method for accurately diagnosing HCC. When it is difficult to demonstrate non-enhanced MRI of tumors, radiologists can use contrast agents (such as Gd^3+^, Fe_3_O_4_, or FePt) for T1-weighted and T2-weighted imaging remain in the liver for a long time to facilitate diagnosis via MRI. However, it is sometimes difficult for T2-weighted imaging to detect small tumor lesions because the liver tissue may absorb iron ions. This makes early cancer detection a challenging goal. This challenge has prompted current research to create novel nanocomposites for enhancing the noise-to-signal ratio of MRI. To develop a method that can more efficiently diagnose and simultaneously treat HCC during MRI examination, we designed a functionalized montmorillonite (MMT) material with a porous structure to benefit related drugs, such as mitoxantrone (MIT) delivery or as a carrier for the FePt nanoparticles (FePt NPs) to introduce cancer therapy. Multifunctional FePt@MMT can simultaneously visualize HCC by enhancing MRI signals, treating various diseases, and being used as an inducer of magnetic fluid hyperthermia (MFH). After loading the drug MIT, FePt@MMT-MIT provides both MFH treatment and chemotherapy in one nanosystem. These results ultimately prove that functionalized FePt@MMT-MIT could be integrated as a versatile drugs delivery system by combining with MRI, chemotheraeutic drugs, and magnetic guide targeting.

**Supplementary Information:**

The online version contains supplementary material available at 10.1186/s12951-021-01052-7.

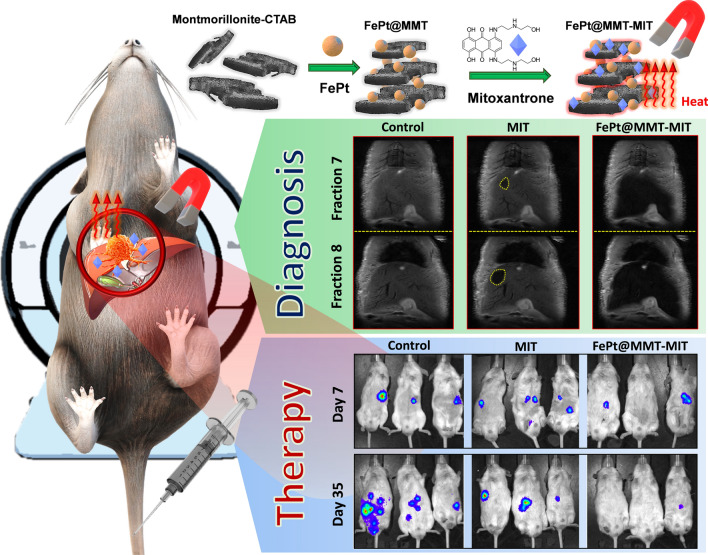

## Introduction

High malignancy of Hepatocellular carcinoma (HCC) ranks second in the fatality rate in Asia [1–3], including Taiwan, Japan, and China. It has become one of the top ten leading cancers in the world [9]. The incidence of HCC in Western countries is relatively low [10]. Most patients with HCC appear to be clinically healthy, with almost no relevant signs or symptoms, while having large tumors in the liver [11–13]. Because of the anatomic location of the liver, it is impossible to have a palpable tumor lump during palpation [18]. However, when HCC is diagnosed, the clinical course of patients may deteriorate very quickly. For patients who have a high risk for developing HCC, such as carriers of Hepatitis B virus (HBV) and Hepatitis C virus (HCV), liver cirrhosis caused by any etiologies, and steatotic liver changes, screening and early detection of HCC is the most effective and possible curative strategy in treating HCC [19–21]. Without regular screenings, the potential threat caused by tumor tissue can be easily overlooked [22]. Among the many screening and diagnostic methods, the advantage of magnetic resonance imaging (MRI) is that it does not generate any ionizing radiation [23–25]. Instead, it uses the principle of nuclear magnetic resonance (NMR) to generate images under a magnetic field. MRI can provide high-resolution and high-quality images of the liver. To improve the ability to detect HCC in the liver, the addition of superparamagnetic iron-based nanoparticles can be used as a contrast developer to display a high-intensity mode on T2-weighted MRI images [26–28].

In the past decades, iron oxide-based T2-weighted contrast agents have been used in clinical trials or been approved by the U.S. Food and Drug Administration [31]. Besides iron oxide nanoparticles, iron platinum nanoparticles (FePt NPs) also enable accurate MRI detection, which features the generation of magneto-caloric energy, enabling further use in cancer-related applications [8, 32, 33]. However, because liver tissue actively absorbs iron ions, it is difficult to detect a tumor *in vivo* imaging and makes it difficult for T2-weighted imaging to detect HCC with a general iron-based contrast agent [34]. Thus, the optimization of contrast medium, such as the superparamagnetic iron-based nanomaterial, has become a critical issue when used as a contrast medium for MRI to detect HCC accurately [38]. Even if the relative position of cancer cells can be aware by the dark field changes obtained by T2-weighted MRI detection; however, insufficient contrast between dark colors may cause interpretation errors, especially in the liver organ [39]. This problem has prompted scientists to consider creating new MRI contrast nanocomposites [40, 41]. To improve the efficiency of magnetic carriers, related medical research using multifunctional composite nanometers has successfully developed many applicable carriers for imaging, drug-loading, and calibration purposes [42–44]. For instance, Yang *et al.* used seed-mediated nucleation to construct MnO and form heterogeneous nuclei on the corners and edges of FePt NPs [6]. The authors then used the solvent exchange to coat the FePt@MnO particles to enhance the signal of MRI. Emo *et al.* used a double iron-based model, FePt@Fe_3_O_4_ nanoparticles, to treat leukemia [4]. This nanocomposite with PEG chains can increase the internalization ratio of nanoparticles, prompt the therapeutic effect, improve the image contrast of tumors, and induce tumor cell death through local temperature increases. Here, we have compiled a table to compare the difference between the previous study and this research work (Table 1).

**Table 1:** Various FePt nanoparticle composite materials are used to enhance their biological applications in MRI

Nanocomposite	Particle size	Saturation magnetization (M_s_)	*r*^*2*^ relaxivity (Tesla)	Cell types	Refs.
FePt-Fe_3_O_4_ nanocubes	10–13 nm	26.5 emu·g^−1^	n/a	THP-1 cell line	Emo et al*.* [4]
FePt@Kaolinite nanocomposites	100 ± 10 nm	25.86 emu g^−1^	17.18 mM^−1^ s^−1^ (4.7 T)	HepG2 cell line	Chan et al*.* [5]
FePt@MnO nanoparticles	8 ± 1 nm	n/a	8.14 mM^−1^ s^−1^ (3 T)	HepG2 cell line MCF-7 cell line	Yang et al*.* [6]
FePt@iron oxide nanoparticles	14.7 ± 1.1 nm	471 emu cc^−1^	360 mM^−1^ s^−1^ (4.7 T)	KB cell line	Yang et al*.* [7]
FePt/SiO_2_/Au nanoparticles	40 nm	1140 emu cm^−3^	47 ± 3 mM^−1^ s^−1^ (2.35 T)	RT4 cell line	Kostevšek et al*.* [8]
FePt@hollow mesoporous silica nanospheres	100–150 nm	9.4 emu g^−1^	46.2 mM^−1^ s^−1^ (9.4 T)	HeLa cell line	Lin et al*.* [14]
FePt@mSiO_2_	87.6 ± 6.5 nm	n/a	7.173 mM^−1^ s^−1^ (0.47 T)	HeLa cell line	Chen et al*.* [15]
FePt-CdS nanoprobe	9–11 nm	n/a	538.1 s^−1^ mg^−1^ mL	RAW 264.7 macrophage cell line	Jha et al*.* [16]
FePt/graphene oxide nanoassemblies	100 nm	4.10 emu g^−1^	12.425 mM^−1^ s^−1^ (3 T)	MCF-7 cell line normal L02 cell line	Yue et al*.* [17]
FePt–Fe_3_O_4_ core–shell nanoparticles	8 nm	23.1 emu g^−1^	131.5 mM^−1^ s^−1^ (9.4 T)	n/a	Kim et al*.* [29]
FePt@Fe_2_O_3_ core–shell magnetic nanoparticles	5 nm	n/a	91.9 mM^−1^ s^−1^ (3 T)	KB cell line HeLa cell line	Liu et al*.* [30]
FePt@SiO_2_ nanoparticles	48.98 ± 6.41 nm	~ 10 emu g^−1^	102.3 mM^−1^ s^−1^ (3 T)	HeLa cell line	Lai et al*.* [35]
Si@FePt- Fe_3_O_4_ nanoparticles	~ 100 nm	~ 0.09 emu cm^3^/mol	~ 20 mM^−1^ s^−1^	n/a	Malvindi et al*.* [36]
FePt@Fe_2_O_3_ nanoparticles	8 nm	24.8 emu g^−1^	3.462 (μg/mL)^−1^ s^−1^ (7 T)	HeLa cell line	Gao et al*.* [37]
FePt@ Montmorillonite nanocomposites (This work)	150–200 nm	24.54 emu g^−1^	41.835 mg^−1^ s^−1^ mL (7 T)	SKHep1 cell line Mahlavu cell line	-

Therefore, montmorillonite (MMT) can be used as a magnetic composite material for absorbing FePt NPs and optimized for MRI-related applications. The use of MMT combined with magnetic materials has been proven to increase the saturation magnetization (Ms) and enhance the contrast signal of T2-weighted MRI [45–47]. FePt NPs included in MMT-modified cetyltrimethylammonium bromide (CTAB) have been synthesized through a simple process using the one-pot method. Triethylene glycol (TEG) functions as both a solvent and reductant, whereas MMT-modified CTAB provides anchoring sites for the nucleation growth of FePt NPs. After the CTAB was calcined and removed, FePt@MMT had a more substantial magnetic flux than FePt, making this nanocomposite a new type of T2-weighted MRI contrast agent. Based on the analytical results, it can be observed that the Ms of FePt NPs was only 14.67 emu/g. However, the Ms of optimized FePt@MMT nanocomposites (FePt@MMT/10.5%) increased to 24.54 emu/g (175% higher than FePt), which can be applied for achieving in situ MRI diagnosis. Due to the inefficient suppression of tumor tissue with a single treatment, cocktail therapy has become the current standard for cancer treatment. The heating response of hydrophilic FePt@MMT nanocomposites was measured using a high-frequency heater, which demonstrated that this nanocomposite could raise the water to 60 degrees, thereby killing cancer cells through magnetic fluid hyperthermia (MFH). Compared with simply using FePt NPs, the spatial limitation of the layer space can further enhance the magnetocaloric effect to generate more heat energy. Moreover, porous MMT layers can simultaneously be used as a drug-loading platform and capture chemotherapeutic drugs, such as mitoxantrone (MIT), to suppress central tumor growth [48–50].

In this study, CTAB enabled MMT to form a layered structure. The final sheet-like MMT was used to obtain a uniform nanoscale in processing ultrasonic waves and thermal environment and react with the FePt NPs to generate FePt@MMT; a layered MMT optimized the related magnetic properties to access material with chemotherapy and magnetic fluid hyperthermia and rustic HCC progression. With magnetocaloric effect enables FePt@MMT to load MIT to form FePt@MMT-MIT, which is used as an MFH/chemotherapy drug for cancer treatment. Accompany the use of MMT and the establishment of composite magnetic nanoplatforms to absorb MIT and FePt NPs; it is also expected to improve MRI resolution and cancer-related effects.

## Materials and methods

### Materials

All chemicals were used without purification. Montmorillonite K 10 (MMT) and oleic acid were purchased from Tokyo Kasei (Tokyo, Japan). Cetyltrimethylammonium bromide (CTAB, 99%), mitoxantrone hydrochloride (MIT, 98.0–102.0%), tetraethylene glycol (TEG, C_6_H_14_O_4_), iron(III) acetylacetonate (Fe(acac)_3_, 99.9+%) and platinum(II) acetylacetonate (Pt(acac)_2_, 99%) were obtained from Sigma-Aldrich (Saint Louis, MO, U.S.). Ethanol (C_2_H_5_OH, 99.8%) was purchased from Nippon Kayaku (Tokyo, Japan). Tetraethylene glycol (C_6_H_14_O_4_) was obtained from Alfa Aesar (Haverhill, MA, USA). The minimum essential medium (MEM), Dulbecco's modified eagle medium (DMEM), fetal bovine serum (FBS), and penicillin-streptomycin-glutamine (PSG) supplement for the culture medium were purchased from Gibco (Waltham, MA, USA).

### Preparation of CTAB-modified MMT

First, 0.5 g of MMT powder and CTAB powder (0.25 g) were weighed, 30 mL of deionized water was added as the reaction solvent, and then the mixture was placed in a 50 mL three-necked round bottom flask. The fin-shaped stirring magnet was inducted into the three-necked round bottom of the flask, and an experimental device was set up. An appropriate amount of silicone oil was added to the glass sleeve where the temperature probe is placed to help conduct the heat source so that it is easy to measure and control the temperature change. The temperature rise began, where the heating rate was set to 5 °C /min and heated to 80 °C. When the system temperature rose to 80 °C and was maintained for 12 h, the heating source was turned off and cool to room temperature naturally. Deionized water was added and centrifuged (6000 rpm, 30 min) to perform the purification procedure and then repeated. The remaining surfactant was removed five times, and the purified sample was vacuum dried to obtain a white powder of CTAB-modified MMT.

### Synthesis of FePt NPs

Fe(acac)_3_ (0.75 mmol) and Pt(acac)_2_ (0.5 mmol) were used as the reaction precursors; then, we added tetraethylene glycol (30 mL) to the reaction solvent in a 50 mL three-necked round bottom. Argon was introduced into the three-necked round bottom flask for 30 minutes as a gas replacement. Next, a heating step was performed, setting the heating rate to 5 °C per min until it reached 200 °C. When the temperature reached 200 °C, it was held for 1 hour, and then we added oleic acid (1 mL) surfactant and continued to maintain this temperature for 30 minutes. After heating to 300 °C at a heating rate of 5 °C per min, the reaction was refluxed for 1 h. During the heating process, the color of the solution was observed to change from blackish-red to black, indicating that FePt NPs had formed. Then, ethanol was added, followed by centrifugation at 6000 rpm for 1 hour, and the procedure for particle purification was conducted. The particles were precipitated due to the increase in the polarity of the solvent, and the purified FePt NPs were washed five times, after which the purified FePt NPs were dried. The black powder was then collected to obtain a hydrophilic phase of FePt NPs.

### Synthesis of FePt@MMT nanocomposites

The use of chemical reduction in a one-pot method to prepare cetyltrimethylammonium bromide-modified MMT and ferroplatin nanoparticle nanocomposites was researched. After surface modification of MMT, TEG was used as the reducing solvent, and an iron precursor, platinum precursor, and modified MMT were added simultaneously using a simple one-pot method to synthesize the nanocomposites.

### Synthesis of the FePt@MMT-MIT drug-carrier nanoplatforms

First, 20 μg/mL of MIT was added to 1 mg of FePt@MMT (with and without CTAB modification) overnight and removed via magnetic separation at room temperature for 10 minutes to obtain FePt@MMT-MIT. Moreover, we chose methylene blue (MB), brilliant green (BG), and Congo red (CR) as the control groups to prove the adsorption ability of FePt@MMT. The efficiency and percentage of dye adsorption can be calculated using Eq. 1.

1 $${Q}_{e}=\left({C}_{0}-{C}_{e}\right)V/m.$$

Next, we calculated the adsorption capacity Q_e_ of the composite nanomaterial, where C_0_ is the initial dye concentration (mg/L), C_e_ is the concentration at time t (mg/L), V is the volume of the dye solution (L), m is the weight (g) of the adsorbent, and the Q_e_ unit is expressed by the weight (mg/g) of the adsorbed dye.

### Magnetic vibration and MRI analysis

A vibrating sample magnetometer (VSM) can measure the magnetic field and the moment of the material by placing the samples at a fixed frequency in the direction of a certain vertical magnetic field. In terms of magnetic measurements, a vibrating sample magnetometer is used to measure the magnetic properties of the Fe-Pt-Fe nanoparticles. The measurement conditions were at room temperature, and an external magnetic field of 16000 Oe to -16000 Oe was applied. It was analyzed whether the material is suitable for an MRI imaging agent using a magnetic resonance imager. In this experiment, the size of the nuclear magnetic resonance magnetic field was 7T, samples of different concentrations were prepared in a 0.5% agar peptizer, and the spin-spin relaxation time (T2) of the material was measured using a nuclear magnetic resonance instrument. The material's transverse relaxation rate (r2) was obtained by plotting the relaxation rate (1/T2) against the ion concentration. For *in vivo* MRI T2-weighted imaging (*In vivo* BRUKER Biospec 4.7T 40-cm bore horizontal MRI system, Karlsruhe, Germany), we set the echo spacing = 8 ms, numbers of TEs = 15, FOV = 6.5 × 6.5 cm^2^, matrix size = 256 × 256, NEX = 2, and the concentration of FePt@MMT added was from 0.1 to 1.6 mM.

### Magnetic fluid hyperthermia (MFH) analysis

A high-frequency heater is a technology that uses MFH electromagnetic induction to achieve heating. This study used CEIA's Power Cube 32/900 (CEIA, Viciomaggio, Italy) and Luxtron's I652 fiber thermometers (Rochester, NY, U.S.). A high-frequency heater can be used to determine whether the material can heat the magnetic nanoparticles in a short amount of time under the interaction of an external magnetic field. The high-frequency heater used in this research has an output frequency of 800 kHz and a magnetic field strength of 3.8 kA/m. The sample was dispersed in deionized water at a ratio of 6 mg/mL. We then discussed the heating effect of the prepared magnetic nanocomposite material, calculated the slope of the temperature change recorded in a certain period, and then used Eq. 2 to calculate the magnetic nanocomposite material heating capacity (SAR).

2 $$SAR=C\left(\frac{\Delta T}{\Delta t}\right)\frac{1}{{m}_{FePt}}$$

where C is the weight-weighted average of the specific heat capacity of magnetic NPs and water, ΔT/Δt is the relationship between the temperature change and time change, and mFePt is the magnetic NPs' weight. Its unit is the number of heating watts per unit weight (W/g). The heater has an output frequency of 800 kHz and a magnetic field strength of 3.8 kA/m. All samples were dispersed in DI water at a ratio of 6 mg/mL.

### Potentiostat electrochemical detection

The potentiostat used in this experiment was CHI627E from CH Instruments. The electrochemical properties of FePt NPs and the different ratios of FePt/MMT-CTAB composite materials were measured via cyclic voltammetry (CV) curves. This study tested the characteristics of nanocomposites used in methanol fuel cells in a mixed solution of 1 M methanol and 1 M sulfuric acid. An electrochemical three-electrode system was used. The reference electrode was an Ag/AgCl electrode, and the counter electrode was a platinum wire. 3 mg of the sample were mixed with 10 μL of Nafion in 1.5 mL of ethanol, and then ultrasonic vibration was used for 30 minutes to disperse and obtain a liquid catalyst. Three microliters of the prepared liquid catalyst were dropped onto the surface of the glassy carbon electrode and placed on the glassy carbon electrode surface, using oven drying as a working electrode. The FePt NPs and the different ratios of FePt/MMT-CTAB composite materials were measured. The scanning interval was between -0.2 V and 1.2 V. The platinum in FePt NPs, and FePt/MMT-CTAB acts as a catalyst in an environment containing methanol, making an oxidation peak. A reduction peak appears in the cyclic voltammetry curve, respectively.

### In vitro cell viability and cytotoxicity analysis

This study selected the Mahlavu and SKHep1 HCC cell lines as the observation objects of material biocompatibility. These two HCC cell lines have been confirmed by orthotopic transplantation to establish observable animal models in the NOD scid gamma (NSG) mouse system. Moreover, the 293T was chosen as the normal cell line for further evaluation. MEM was added to the pituitary fluid to maintain the growth of the Mahlavu and SKHep1 HCC cell lines, and DMEM was added to support the development of the 293T. In addition, MEM and DMEM with 1% PSG were mixed with 10% FBS as the culture solution for the HCC and normal cell lines. These cells were then cultured at 37 °C and 5% carbon dioxide. Approximately 2,000 cells per well were cultured in 96-well plates for 12 h, while nanocomposites of 3, 9, 27, 81, and 250 μg/mL were added to the individual wells for 48 h. The cell dye Alamar Blue was added similarly. Cell staining was performed, and the fluorescence intensity of Alamar Blue (OD 570 nm and 600 nm) was detected using a fluorophore to quantify the cell viability and cytotoxicity.

### Cellular uptake and localization analysis

Approximately 20,000 Mahlavu and SKHep1 cells per mL were plated on a well plate slide for 12 h, while 250 μg/mL FePt@MMT-MIT was added to the culture at 37 °C with 5% carbon dioxide for 12 h. The cells were then washed with 10 mM phosphate-buffered saline (PBS; pH 7.4) and fixed in 4% paraformaldehyde fixative (paraformaldehyde) to maintain their intrinsic form. Subsequently, the nuclear stain DAPI was added for nuclear staining. After 5 min of incubation, the dye was removed and observed via laser scanning confocal microscopy (LSCM). The stained nucleus can be excited using a 408 nm UV laser, while the emission image can be detected at 450–500 nm. The MIT drugs loaded in FePt@MMT-MIT can also be excited using 648 nm orange to red light, while its emission is detected by red fluorescence at 695 nm.

### In vivo therapeutic effect

The animal experiments were approved by the Institutional Animal Care and Utilization Committees of Academia Sinica (IACUC NO. 18-03-1202). Our study was performed according to the guidelines of the U.S. National Institutes of Health and the recommendations of the committee on animal research at our institution. Therefore, the protocol was approved by the local institutional review committee on animal care. The Mahlavu and SKHep1 cell lines were cultured in MEM with 10% FBS and 1% PSG at 37 °C in an environment containing 5% CO_2_. Next, 6-week-old male NSG mice were chosen for the experimental model. Briefly, 2 × 10^4^ Mahlavu and SKHep1 cells in 25 µL of PBS were mixture with matrix gel with the ratio of 1:2 and orthotopically injected into the liver tissue after being anesthetized by isoflurane. This study was performed using 32 mice to evaluate two types of tumor growth after several weeks (above 5E5 average radiance photon numbers). To assess the effectiveness of *in vivo* MIT (chemotherapy) and MFH, we measured the tumor size by IVIS every week. We compared four groups: the control group (n = 8), the MIT treatment group (MIT only, n = 8), the MFH treatment group (with FePt@MMT but without MIT loading, n = 8), and the MFH + MIT group (with FePt@MMT-MIT, n = 8).

### Tissue staining sections

After that, all tumors were collected after the fifth week. The tumor sections were formalin-fixed and paraffin-embedded. The cross-sections of the tumors were stained using H&E. Briefly, and the units were first dewaxed in a 60 °C oven, deparaffinized in xylene, and rehydrated in graded alcohol. We dissolved 50 g of aluminum potassium sulfate in 1000 mL of distilled water. When the aluminum potassium sulfate was wholly dissolved, we added 1 mg of hematoxylin. When the hematoxylin was utterly dissolved, we added 0.2 mg of sodium iodate and 20 mL of acetic acid. We then brought the solution to a boil, allowed it to cool, and filtered it for use in staining, followed by observing the tissues using a Leica Aperio AT2 scanner.

## Results and discussion

### Materials identification

This study aimed to prepare a new T2-weighted MRI contrast agent with good biocompatibility and high saturation magnetization. The layer structure can limit the space of the magnetic material and optimize magnetic efficiency. For this reason, coating layered ceramic materials, such as MTT with magnetic nanoparticles (such as FePt or iron oxide NPs) can increase the saturation magnetization and reduce the coercive force. The primary reason is that the NPs are subjected to uniaxial compression stress at a vertical angle to the MMT layer. The resulting mechanical stress causes the magnetic dipoles of the NPs to rearrange and makes them parallel to the direction of the compressive stress. Therefore, the saturation magnetization follows the magnetization direction and enhances the magnetic flux, as shown in Additional file 1: Figure S1a. However, as a candidate for the T2 contrast agent, iron oxide has the drawback of being prone to corrosion and degradation in unpredictable biochemical environments. FePt NPs have good biocompatibility, high chemical, and physical stability, and a low cost to increase the saturation magnetization via co-synthetic nanocomposites. They can be integrated with MMT to achieve a high adsorption performance of the drug. This study first processed bulk MMT material via CTAB and ultrasound to produce a layered structure. To observe the surface morphology of CTAB-modified MMT, scanning electron microscopy (SEM) was used to evaluate the form of the particles (Additional file 1: Figure S1b). It was determined from the low-magnification images that the MMT material is evenly distributed in a layered structure after modification. Since the CTAB polymer needs to be removed from MMT, the calcining process converts CTAB to CO_2_. After an evaluation with thermogravimetric analysis (TGA), it was confirmed that CTAB was dismissed at 500 degrees (Additional file 1: Figure S1c). An X-ray powder diffractometer (XRD) was used to analyze the crystal structure of the MMT and CTAB-modified MMT, as shown in Additional file 1: Figure S1d.

The measured data were then compared with the MMT standard map (JCPDS 29-1498). The comparison showed that the MMT modified by CTAB has higher characteristic diffraction peaks than the pure MMT at 8.9, 19.6, 34.9, and 61.7 degrees. This indicated that the MMT modified by CTAB is superior in crystallinity to pure MMT. In addition to the diffraction peaks of MMT, various other diffraction peaks can be seen, caused by impurities (quartz, muscovite, and feldspar). Functional groups on the surface of MMT and CTAB-modified MMT were analyzed via Fourier transform infrared spectrometry (FTIR). As seen in Additional file 1: Figure S1e, after the surface modification of CTAB, the stretching of the Si-O bond results in a change in the absorption peak at 1033 cm^-1^, and the absorption band is stretched at 2820−2960 cm^-1^, corresponding to the C–H bond. The peak at 1440 cm^-1^ on the MMR corresponds to the bending of the C–H bond, which indicates that long-chain alkyl groups are present in the CTAB of the CTAB-modified MMT.

**Fig. 1:**
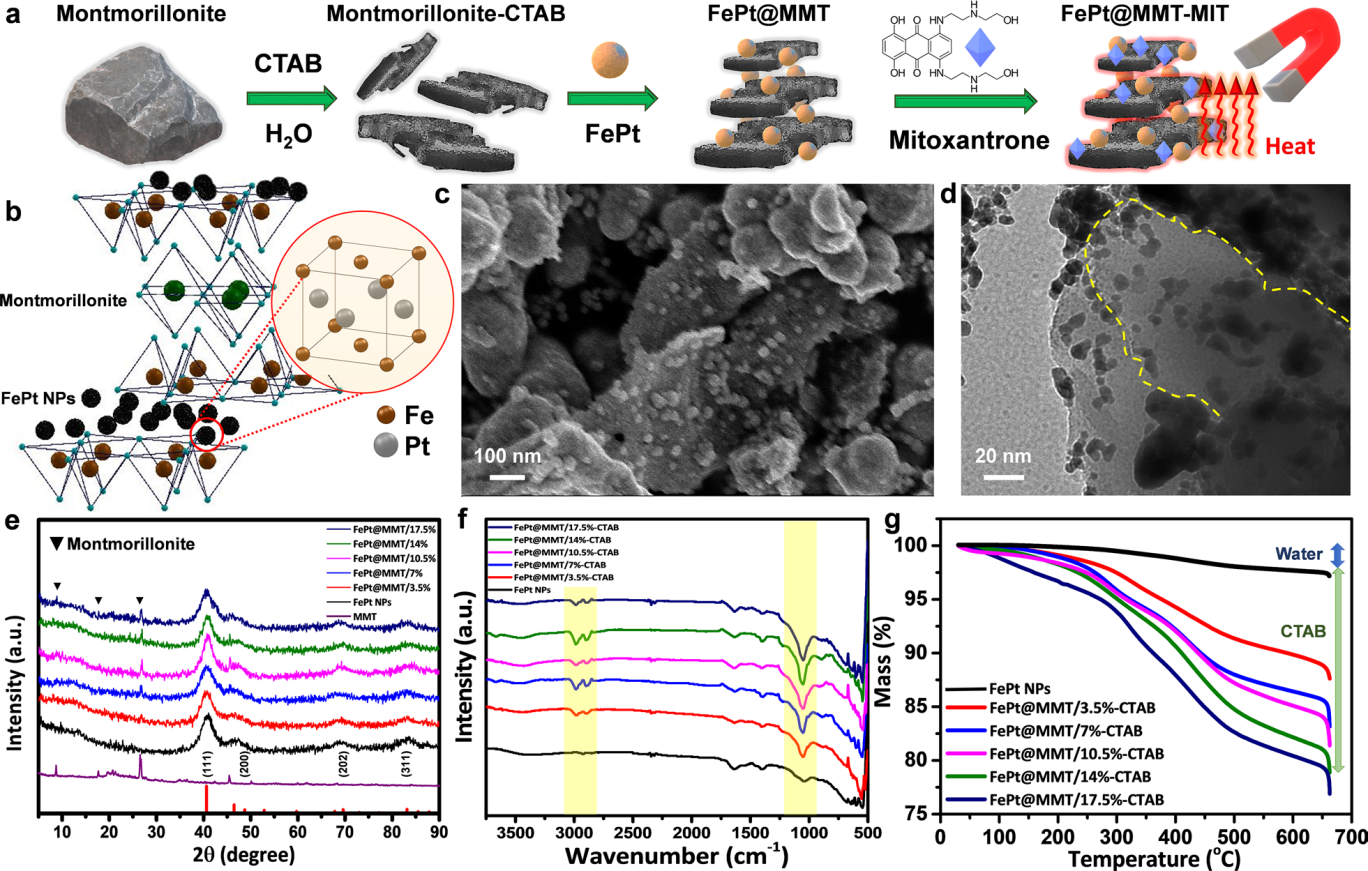
Identification of FePt NPs and the FePt@MMT derivation. **a** Schematic diagram illustrating how the FePt@MMT-MIT be prepared. **b** The crystal structure of the FePt@MMT nanocomposite. TEM images of **c** FePt@MMT NPs and **d** the relative lattice distance of FePt NPs in FePt@MMT **e** XRD data of different MMT concentration ratios of FePt@MMT. **f** FTIR and **g** TGA data show the functionalized CTAB molecules and the sintering temperature of CTAB, respectively

This research uses a simple, easy-to-implement, safe and effective method to produce a FePt nanocomposite material with magnetic and adsorption properties. Since more than half of the biological components in the body are water, this work is also intended to improve the dispersibility and biocompatibility in a hydrophilic environment. The FePt nanocomposites for the experiment were prepared using a one-pot synthesis. The one-pot synthesis method is chosen because it is simple to use, has a large output, and is hoped to produce crystalline materials at low temperatures. It is thus expected that a multifunctional nanocomposite material with high adsorption and magnetic properties can be prepared (Fig. 1a and b). First, Fig. 1c and d show the structural analysis of FePt@MMT using SEM, Transmission Electron Microscopy (TEM), and scanning TEM (STEM). An additional STEM gif. file shows this in more detail about the structure of FePt@MMT [see Additional STEM file 1]. It can be seen that FePt NPs are sandwiched between the layered structures of the MMT. To optimize the concentration gradient of MMT modified for the FePt NPs, we chose 3.5, 7, 10.5, 14, and 17.5% for further evaluation. The crystal structure of the FePt NPs was analyzed using X-ray powder diffraction (XRD), as shown in Fig. 1e, compared to MMT and its standard map (JCPDS No. 43-1359). The prominent diffraction peaks of FePt NPs are located at 41.05, 47, 71, and 84 degrees with orientations of (111), (200), (202), and (311), respectively. Due to the lack of external energy, synthesized FePt NPs cannot overcome the activation energy of the ordered phase transition. This indicates that a chemically disordered fcc Fe-Pt structure with a (111) orientation is formed. With varying amounts of MMT in the FePt@MMT nanocomposite, the diffraction peak of MMT was observed at 8.9, 19.6, and 23.9 degrees at MMT concentrations greater than 7%, and it was concluded that the higher the proportion of MMT, the higher the peak intensity. FTIR was then used to analyze the surface functional groups of the FePt@MMT-CTAB nanocomposites. As shown in Fig. 1f, the functional groups are not much different from pure FePt NPs or CTAB-modified MMT. The change in the absorption peak at 1033 cm^-1^ is the stretching of the Si-O-Si bond. Thus, as the proportion of CTAB-modified MMT increases, the absorption peak intensity also becomes more vigorous. The thermal stability of the FePt@MMT-CTAB nanocomposite was analyzed via TGA. As shown in Fig. 1g, the weight loss of FePt@MMT-CTAB nanocomposites before 100 °C is mainly due to the evaporation of physically adsorbed water, while the weight loss at 240−400 °C increases with an increase in the proportion of MMT-modified CTAB. From the results, it can be inferred that the cleavage of the surfactant CTAB mainly causes the difference in weight loss. According to previous experimental results, the weight loss at 240−400 °C also includes the decomposition of surface functional groups modified by the surfactant oleic acid.

To confirm that the magnetization ability of the FePt material will not be affected by the interlayer of MMT to produce a shadowing effect, the catalytic efficiency of Pt can act as a catalyst in the presence of methanol, which causes reduction and oxidation peaks in the cyclic voltammetry (CV) curve according to Eq. 3.

3 $$\begin{gathered} {\text{Oxidation}}:{\text{~Pt}} - \left( {{\text{CH}}_{3} {\text{OH}}} \right)_{{ADS}} \to {\text{Pt}} - \left( {{\text{CH}}_{2} {\text{OH}}} \right)_{{ADS}} + {\text{H}}^{ + } + {\text{e}}^{ - } \hfill \\ {\text{Pt}} - \left( {{\text{CH}}_{2} {\text{OH}}} \right)_{{ADS}} \to {\text{Pt}} - \left( {{\text{CHOH}}} \right)_{{ADS}} + {\text{H}}^{ + } + {\text{e}}^{ - } \hfill \\ {\text{Pt}} - \left( {{\text{CHOH}}} \right)_{{ADS}} \to {\text{Pt}} - \left( {{\text{COH}}} \right)_{{ADS}} + {\text{H}}^{ + } + {\text{e}}^{ - } \hfill \\ {\text{Pt}} - \left( {{\text{COH}}} \right)_{{ADS}} \to {\mathbf{Pt}} - \left( {{\mathbf{CO}}} \right)_{{\user2{ADS}}} + {\text{H}}^{ + } + {\text{e}}^{ - } \hfill \\ {\text{Reduction}}:{\text{~}}{\mathbf{Pt}} - \left( {{\mathbf{CO}}} \right)_{{\user2{ADS}}} + {\text{OH}}^{ - } \to {\text{Pt}} + {\text{CO}}_{2} + {\text{H}}^{ + } + {\text{e}}^{ - } \hfill \\ \end{gathered}$$

An oxidation peak presence of methanol at 0.6~0.75 V, and methanol can then generate a Pt-CO bond on the surface after catalysis via Pt. For the induced potential by reduction, the peak of CO reduction to CO_2_ desorption occurs at 0.35~0.5 V. As seen in Additional file 1: Figures S2a–S2f, the current density of the sample after adding MMT increases proportionally with the amount added. After adding MMT, the relative Pt content is presumed to be less than the initial concentration, thus resulting in a relatively lower density of the products. A potentiostat electrochemical analysis confirmed that different concentrations of MMT did not affect the reaction ability of FePt (Additional file 2).

### Magnetic characteristics measurement of FePt NPs and FePt@MMT

**Fig. 2:**
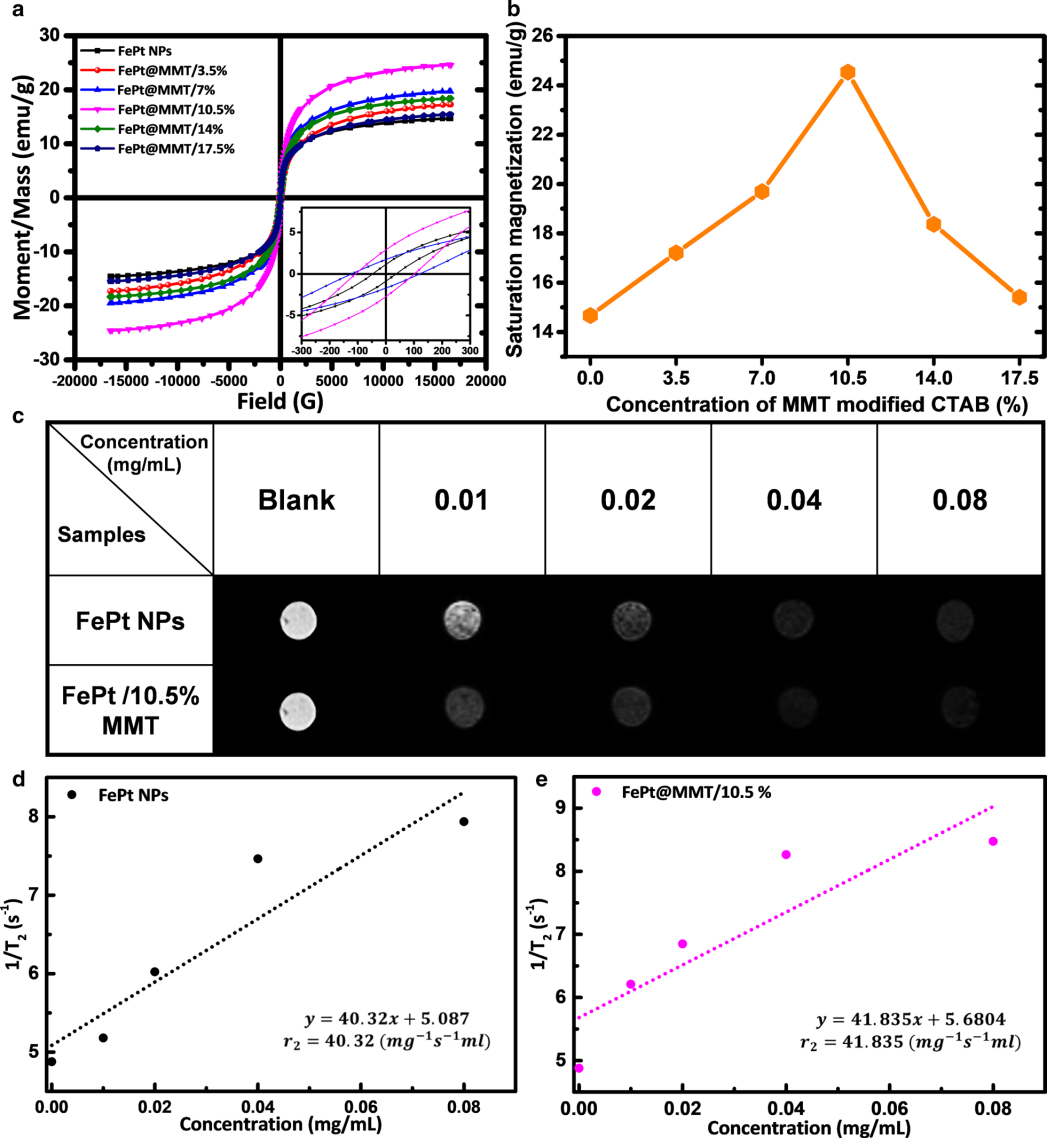
The magnetic physiognomies and composition differences between FePt NPs and FePt@MMT. **a** The related magnetic properties were conducted with a vibrating sample magnetometer and the hysteresis curves analysis of FePt and FePt@MMT. Its magnetic properties were 16,000 Oe to − 16,000 Oe. Hysteresis curves of different proportions of MMT-modified CTAB synthesis series were added. Its magnetic behavior is paramagnetic. **b** VSM quantized values. The inflection point of FePt@MMT was about 10.5%. **c** The T2-weighted MRI contrast images of FePt and FePt@MMT/10.5%. MRI-related analysis, including T2-weighted imaging and 1/T2 linear regression between **d** FePt NPs and **e** FePt@MMT nanocomposites

The magnetic properties of FePt NPs and FePt@MMT were measured using a vibrating sample magnetometer. The measurement conditions were at room temperature with an external magnetic field of 16000 Oe to -16000 Oe. As shown in Fig. 2a and b, its magnetic behavior is paramagnetic, and the amount of magnetization shows a trend of increasing with the addition of MMT-modified CTAB. The saturation magnetization of the FePt NPs is 14.67 emu/g, which rises to 24.54 emu/g after a combination with a 10.5% MMT sheet (FePt@MMT/10.5%). However, when the proportion of MMT increased to 14%, the saturation magnetization began to decrease. It is speculated that the reason may be that the influence of nonferromagnetic MMT has a higher total weight ratio than FePt NPs, resulting in a decrease in the saturation magnetization of FePt@MMT/14.5% to 18.37 emu/g and FePt@MMT/17.5% to 15.32 emu/g. Analyzing the material with a magnetic resonance imager is suitable for use as an MRI imaging agent. In this experiment, the size of the nuclear magnetic resonance field was 7T, samples of different concentrations were prepared in a 0.5% agar peptizer, and the spin-spin relaxation time (T2) of the material was measured using a nuclear magnetic resonance instrument. The material's transverse relaxation rate (r2) was obtained by plotting the relaxation rate (1/T2) against the ion concentration. The FePt NPs and FePt@MMT/10.5% were first measured at concentrations of 0.01, 0.02, 0.04 and 0.08 mg/mL. As seen in Fig. 2c, the T2-weighted image darkens as FePt NPs increase, which means that the higher the concentration of nanoparticles, the better the contrast imaging effect. Then, T2 was converted to 1/T2. As shown in Fig. 2d, linear regression is performed on 1/T2, and r2 is 41.835 mg^-1^s^-1^mL, which indicates that Feplatin nanoparticles can shorten the transverse relaxation time and reduce the effect of magnetic resonance. The signal intensity can then be used as a contrast agent for MRI. Next, the FePt@MMT/10.5% nanocomposite was measured. The T2-weighted image darkens as the concentration of FePt@MMT/10.5% increases. This means that the higher the concentration of the nanocomposite, the better the contrast effect. Then, T2 was converted to 1/T2, as shown in Fig. 2e. Linear regression was performed on 1/T2, and r2 was obtained as 40.32 mg^-1^s^-1^mL. The r2 value did not change significantly. The phenomena demonstrate although FePt@MMT/10.5% sacrifices the number of magnetic nanoparticles, it is due to the saturation magnetization. This amount is higher than that of FePt NPs, so it will ultimately enhance the T2 imaging effect of MRI.

### Magnetic hyperthermia analysis of FePt NPs and FePt@MMT

**Fig. 3:**
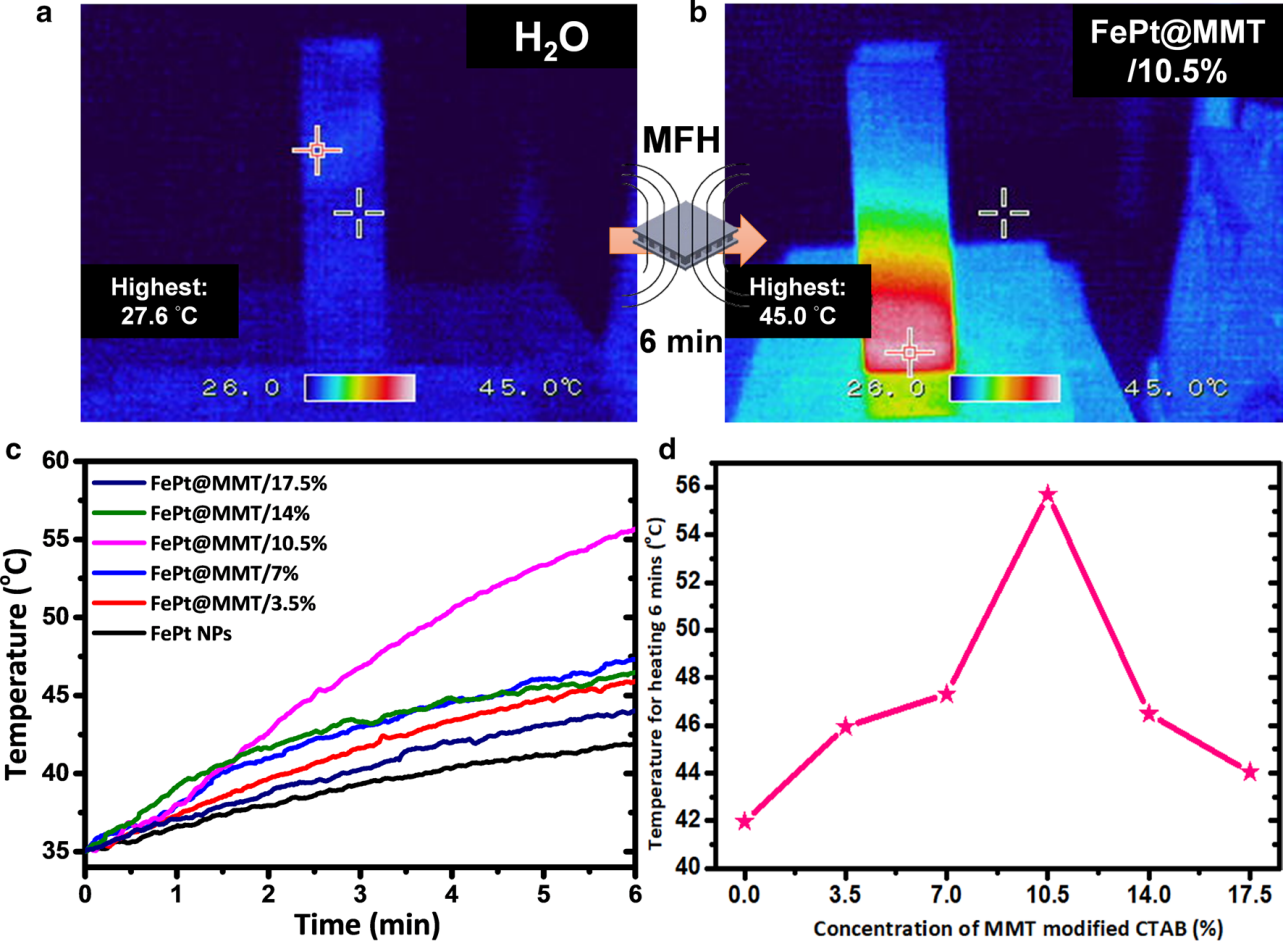
The magnetic fluid thermal image for FePt@MMT. **a**, **b** Temperature-induced analysis on FePt@MMT nanocomposites reveals the related magnetically induced magnetic fluid thermal image, which is captured by an infrared thermal imager **c** Magnetic heating curves and **d** the temperature of FePt NPs or FePt@MMT (the temperature was detected after heating for 6 min)

The high-frequency heater can be used to determine whether the FePt@MMT nanocomposite can be heated up in a short amount of time under the interaction of an external magnetic field to a temperature sufficient to kill cancer cells. The high-frequency heater used in this research has a magnetic field strength of 3.8 kA/m and an output frequency of 800 kHz. FePt@MMT was dispersed in deionized water at a ratio of 10 mg/mL. It can be observed that the heating rate of FePt NPs is the slowest. As the proportion of MMT increases, the heating rate of other FePt@MMT/10.5% nanocomposites also increases significantly, compared with other groups. However, since the detection limit of the thermal imager is only approximately 50 degrees, it cannot match the value obtained by the thermometer in Fig. 3a and b. Based on the thermal analysis of the magnetically processed FePt@MMT/10.5% through a thermal imager, and it can be seen that the temperature inside the cuvette rises sharply. Next, the increased temperature effect of the prepared magnetic FePt@MMT/10.5% is discussed. The slope of the change in the temperature recorded in a certain period is calculated, which is the degree of increased temperature per unit time in Fig. 3c and d. Then, Equation 2 was used to calculate the magnetic nanocomposite material. The heating capacity (SAR) is shown in Additional file 1: Table S1, where it can be seen that the greater the heating rate, the greater the slope of the sample, indicating that it can be heated to a higher temperature at the same time. We can determine that we have successfully prepared a magnetic FePt@MMT nanocomposite material that can quickly heat up to a temperature sufficient to kill cancer cells from the above results. It has a considerable biomedicine potential to kill cancer cells with magnetic hyperthermia.

### FePt@MMT drug loading and evaluation experiments

**Fig. 4:**
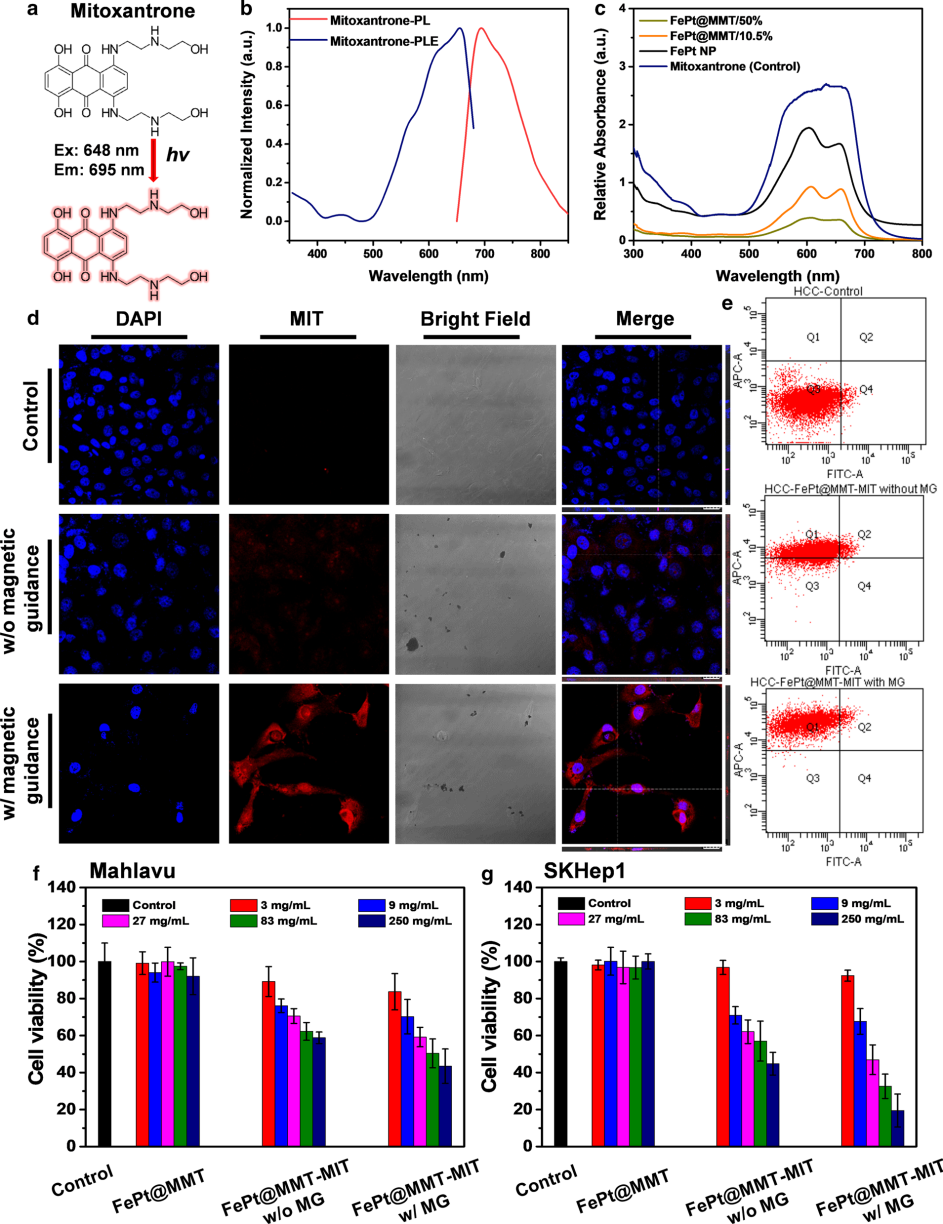
Analysis of the toxicity and viability of nanocomposites to cells. **a** The molecular structure of MIT. **b** PL and PLE of MIT. **c** The drug loading efficiency of FePt@MMT-MIT. **d** The endocytosis efficiency of MIT shows the magnetic permeability effect of FePt@MMT-MIT. The nucleus is shown by DAPI (440 nm) staining; MIT is shown by red fluorescence (695 nm). (scale bar: 50 μm) **e** Fluorescent quantification by flow cytometry. The different kinds of nanocomposites were cultured with **f** Mahalavu and **g** SKHep1 cells for 48 h to assess the cytotoxicity of FePt, FePt@MMT, and FePt@MMT-MIT

An ultraviolet-visible light absorption spectrometer was used to measure the change in different dye concentrations after adding FePt@MMT to judge the ability of the sample to adsorb dyes (Additional file 1: Figure S3a). The drug-carrying capacity of FePt@MMT was first determined through the dye adsorption test. We prepared 20 μg/mL of methylene blue (MB), 50 μg/mL of brilliant green (BG), and 50 μg/mL of Congo red (CR) that all added 1 mg FePt NPs, FePt@MMT/10.5% and FePt@MMT/50%. After shaking and placing the samples at room temperature for 10 minutes, magnetic separation removed the magnetic nanocomposite materials. It can be seen in the experimental results that the pure FePt NPs have almost no adsorption capacity for dyes (Additional file 1: Figures S3b–S3d). In contrast, after adding the MMT layer to each dye, the adsorption percentage reached more than 50%, and the BG reached the highest value of 62.14%. Moreover, the adsorption percentage of FePt@MMT/10.5% reached as high as 65%. The highest is 76.07% of MB, as shown in Additional file 1: Table S2. Compared with pure FePt NPs, MMT has more fragmented and smaller holes, increasing the specific surface area and significantly increasing the efficiency and percentage of dye adsorption. However, suppose too much MMT is added to prepare the FePt@MMT/50% nanocomposites. In that case, the multilayer MMT may overlap to generate a thick clay layer without space interference from FePt particles and make it difficult for the system to carry dyes or other drug cargos (Additional file 1: Figures S3b to S3d). The same experiment was also used to measure the chemotherapeutic drug MIT. MIT is an anthraquinone chemotherapy drug with a dark blue solution and anti-cancer effects on fast-growing and slow-growing malignant tumors. It can treat breast cancer, liver cancer, acute non-lymphatic leukemia, multiple sclerosis, etc. It is combined with prednisone for second-line treatment of hormone-insensitive prostate cancer with distant metastasis. In addition, MIT is a drug with fluorescent characteristics that can absorb light at 648 nm and has an emission wavelength of 695 nm (Fig. 4a and b). An ultraviolet-visible light absorption spectrometer can also obtain data that is consistent with the dye-loading analysis. The absorption efficiency of FePt@MMT/10.5% for MIT is close to 75% (Fig. 4c). Since MIT is the main drug in the subsequent chemotherapy process, we use TGA to assist UV-Vis absorption spectroscopy. Based on Additional file 1: Figure S4a, it can be observed that the weight loss of FePt@MMT is not obvious because there is no drug loading. FePt@MMT-MIT loses about 25% more weight than FePt@MMT, and its thermal sintering curve trend is similar to that of pure MIT. The FePt@MMT-MIT and MIT drug concentrations were adjusted to the approximate total amount through the TGA data. The values of drug release were analyzed by UV-Vis spectrometer at different time points. The releasing curve of MIT, MMT-MIT, and FePt@MMT-MIT have been measured in Additional file 1: Figure S4b. To make sure the porous structure of MMT can cause the release of MIT slower. A slower drug release process helps to allow the drug to accumulate at the target location. In addition, it can be known from the confocal results that FePt@MMT-MIT is affected by magnetic force, and bringing drugs to the cells will cause cell apoptosis, so the number of cells seen under the field of view has decreased. Next, we will analyze the potency of FePt@MMT-MIT at the cellular level.

### In vitro toxicity analysis with drug tracking and cell viability

This experiment was mainly designed to prove whether FePt@MMT-MIT can be guided by external magnetic forces in a short amount of time, with FePt@MMT-MIT entering cells and rapidly accumulating in the cytosol to improve the therapeutic effect. FePt@MMT-MIT was initially added to the cells at a 50 μg/mL concentration in a 12-well dish and immediately guided with a magnetic force. After approximately 12 hours, the cells were removed for observation. As shown in Fig. 4d, we used extra magnetic guidance to help FePt@MMT-MIT accumulate in SKHep1 liver cancer cells, indicating that magnetic induction of MIT drug accumulation can be used as a cumulative drug in specific tissues or a targeted approach for target locations. Moreover, this result was also evaluated by flow cytometry (Fig. 4e). Based on the cell gating to circle the cell region, FePt@MMT-MIT can be guided by a magnetic field to accumulate the drug actively. Here we used two types of liver cancer cell lines to confirm the therapeutic effect of the FePt@MMT-MIT nanosystem, namely, Mahlavu (tends to be malignant) and SK-Hep1 (tends to be benign) as HCC cell line models. As the HCC cell line with the highest metastatic ability, Mahlavu is suitable as a model for establishing orthotopic HCC. However, it may be difficult to achieve complete inhibition during the treatment process because of its high cell metastasis. In contrast, SKHep1, which has a minor tumor tissue, has a better therapeutic effect, which shows that the system can be applied to various HCC cell types and provide sound therapeutic effects. The 293T cell line was chosen as the normal cell control group. We first compared FePt and MMT, which had very low toxicity towards those cell lines (Additional file 1: Figures S5a, S5c, and S5e). After combining these two materials into FePt@MMT nanocomposites with different hybrid ratios, the cytotoxicity remained in a state not significantly increased in either cell line (Additional file 1: Figures S5b and S5d). At the same time, we also separately measured the cytotoxicity of MIT to these three types of cell lines to infer the concentration of IC50. In Fig. 4f and g, the cytotoxicity results of FePt@MMT-MIT on Mahlavu and SK-Hep1 cells were evaluated. Related IC50 of FePt@MMT-MIT was measured by serial dilution, 83–250 mg/mL for Mahlavu and 27–83 mg/mL for SK-Hep1, in which the IC90 results show 250 mg/mL with cell proliferation suppression on SK-Hep1 cells due to it was more benign than Mahlavu.

Moreover, the 293T as the normal control group was also evaluated for the cytotoxicity in Additional file 1: Figure S5f. The normal cell demonstrates more sensitive results after treating with FePt@MMT-MIT. The IC50 effect can be measured at a concentration of approximately 9–27 mg/mL.

### In vivo T2-weight MRI and tumor inhibition analysis

**Fig. 5:**
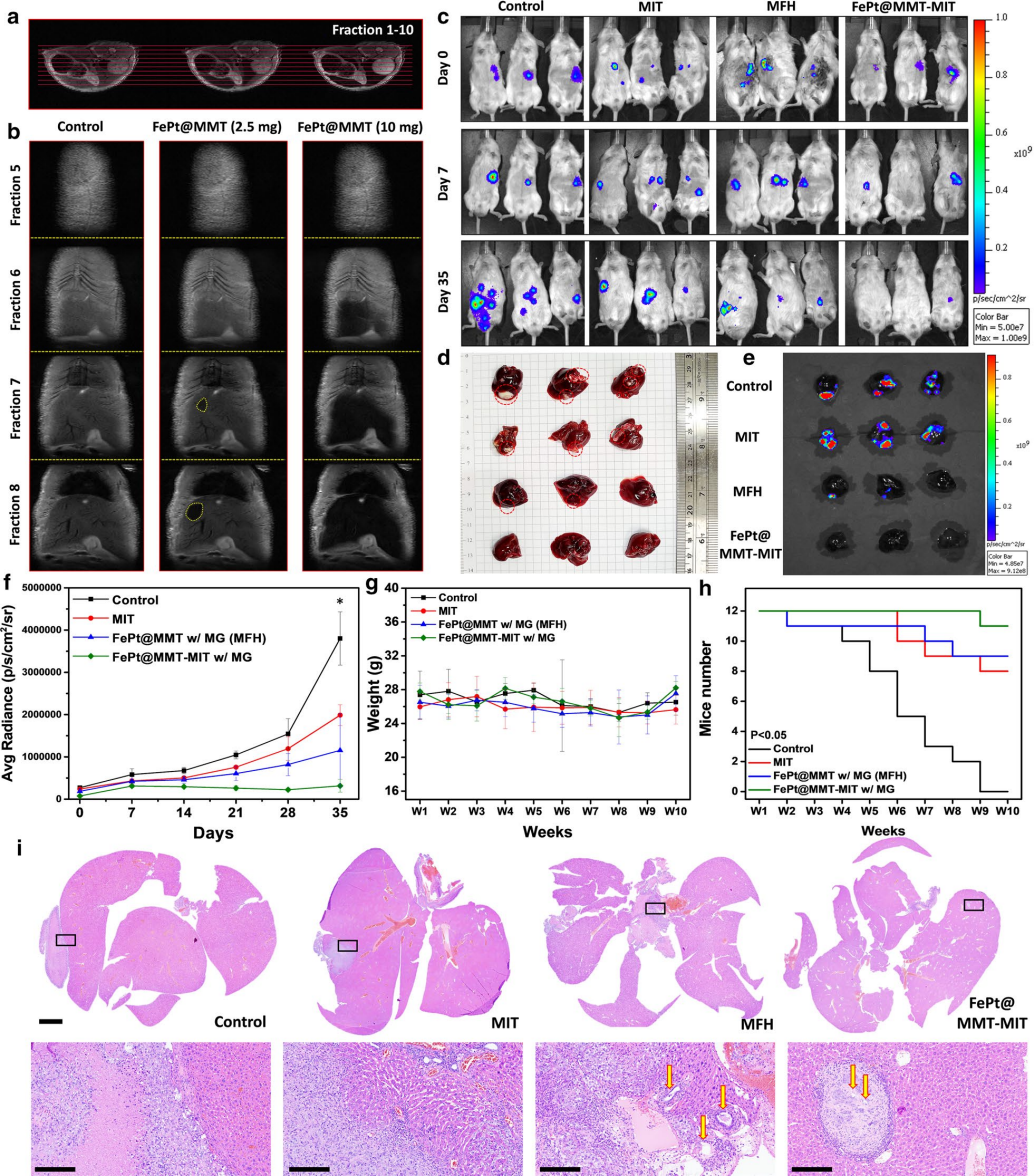
Orthotopic SKHep1 HCC model response to nanocomposites with in vivo I.V. injection. **a**, **b** MRI T2-weighted imaging and transverse images showed the injection results with 100 μL of FePt@MMT in PBS (concentration of 10 mg/mL) into mice. **c** IVIS images show the results of tumors on different nanocomposites, including control, MIT, and FePt@MMT-MIT (The experimental mice were from the same group but arranged differently). The mice liver organs were detected with **d** visual diagrams and **e** IVIS analysis diagrams. Tumor growth assessment for different treatment groups between MFH or MIT treatment, n = 8 per group, including **f** tumor size evaluated from related IVIS images (*p < 0.05 compared with the control group). **g** Bodyweight continued changes of 5-week-old mice and **h** the survival curve of mice with different groups,**i**) and H&E staining result. Related images were visualized from 5× and 40× . (scale bar: 3 μm and 200 nm)

An *in vivo* mouse experiment was conducted at the Genomics Research Center, Academia Sinica. We followed the protocol guidelines with our manager institute, Institutional Animal Care and Use Committee, to apply the protocol with passing number 18-03-1202. We optimized the dose for all different material concentrations of 10 mg/kg in the animal experiments. Only in the T2-weighted MRI data did we use two concentrations to compare the difference in contrast. In the T2-weighted MRI diagnostic test, FePt@MMT was separately injected into mice at different concentrations of 2.5 mg/mL and 10 mg/mL and then induced by a magnetic field to accumulate in the tumor tissue to demonstrate their magnetic characteristics. Clinical 7T MRI imaged mice and different views to analyze the liver contrast image, as shown in Fig. 5a (coronal slice at y = 0) and Fig. 5b (horizontal piece at z = 0). Based on the different segments of the body parts, the results in fraction 7 and fraction 8 demonstrate that FePt@MMT can be easily guided into liver tissue and actively accumulate in the tumor site under magnetic guidance in *in vivo* whole-body images of NOD-SCID mice and can be marked with a yellow circle to indicate the tumor site. After applying FePt@MMT, the T2-weighted MRI signal of the orthotopic HCC tumor became darker. Before injection, the appearance of the tumor area could not be observed under an MRI analysis, which means that the solid magnetic decay was attributed to FePt@MMT, and the dark field imaging of FePt@MMT was more evident at a concentration of 10 mg/mL, indicating that FePt@MMT is a prospective comparative reagent for orthotopic liver MRI contrast.

After confirming the diagnostic efficacy of T2-weighted MRI, we brought FePt@MMT-MIT into mice and evaluated the treatment results. First, we used both the Mahlavu and SKHep1 cell lines have been confirmed to establish observable animal models in the NOD scid gamma (NSG) mouse system. Here we first build to the analysis of the SKHep1 group. Since the orthotopic liver tumor cannot be directly observed, we used the In Vivo Imaging System (IVIS) to evaluate the effect of tumor treatment (Fig. 5c). In the fifth week, the mice were sacrificed to ensure enough mice for statistical calculations, and the liver tissues were excised, as shown in Fig. 5d. In the following animal experiment analysis, we divided all the experimental groups into four groups: the control group, the MIT treatment group (drug only), the MFH treatment group (FePt@MMT with external magnetic treatment but without MIT), and the MFH + MIT group (FePt@MMT under external magnetic treatment). The first is the analysis and tracking of mouse SKHep1 tumor images within five weeks. An excellent therapy via MFH and MIT was demonstrated with a reduction in tumor size and weight by an almost sevenfold decrease can be evaluated under IVIS investigation in Fig. 5c and f (the IVIS average radiance values decrease from 4×10^6^ to 3×10^4^ p/s/cm^2^/sr). After the sacrifice, a significant photon count decrease in the removed livers was distributed in the FePt@MMT-MIT group compared with the control, MFH treatment only, and MIT treatment only groups (Fig. 5e). The MIT+MFH group is more effective in suppressing tumors than the single-drug therapy (MIT and MFH) (about 125 mm^3^). Compared to weight gain in other groups, only the MIT group showed a slight weight loss, which the I.V. injection may cause, and these results support that FePt@MMT-MIT has no significant side effects on mice. (Fig. 5g). Kaplan-Meier survival curves of mice were also evaluated to track the health of the mice *in situ*. After 10 weeks, the control group mice no longer survived without any treatment. Compared with the MIT group, the MFH group and FePt@MMT-MIT group effectively inhibited the growth of SKHep1 cells and reserved the live number of mice from 9 to 12 (Fig. 5h). Furthermore, the liver tumor tissues were image through the H&E staining. It is noteworthy that a clear cavity structure can be observed in the MFH group, which indicates tumor cells may cause necrosis by the heating effect and may be using as an alternative treatment for cancer and make it easier for chemotherapy-related drugs to destroy the remaining cancer cells. The relative curative effect after two weeks of treatment was shown in Fig. 5i. The tumor suppression ability was evaluated by the size of the four groups of tumors.

In addition, we analyzed different kinds of orthotopic liver cancer models, where Mahlavu was another treatment target. We compiled the IVIS system in mice every week and compared the original data of all the data in Additional file 1: Figure S6a to confirm that FePt@MMT-MIT can also be used in the treatment of the Mahlavu cell line. The mouse group using only MIT caused local tumor shrinkage, while FePt@MMT material can effectively rely on the growth of tumors with the effect of MFH. Unlike the previous two groups, the FePt@MMT-MIT group might simultaneously inhibit tumor growth and prevent its metastasis from entering other tissues. We only compare three groups: the control, MIT group, and MFH + MIT group, to evaluate this nanosystem on the Mahlavu cell line. The liver tissues were also removed from the mouse body, and tumor growth was assessed via IVIS (Additional file 1: Figure S6b) and H&E staining (Additional file 1: Figure S6c). Although the image data here shows that the Mahlavu tumor mass on the liver is smaller than SKHep1, the whole mouse IVIS data shows that Mahlavu cells may have spread to various organs. Considering the consistency of results, there is still only taken out the liver tissue for comparison. The results support that the FePt@MMT-MIT nano-targeted drug package can be used as an MFH and MIT-based chemotherapy platform targeting Mahlavu cells. Summarizing the analysis of animal experiments in mice, FePt@MMT-MIT nanoplatform has therapeutic effects on both Mahlavu and SKHep1 cell lines. The experimental results show that FePt@MMT-MIT can almost completely inhibit the growth of the SKHep1 cell line. By contrast, the more malignant Mahlavu is highly metastatic; therefore, even if it has an obvious curative effect in liver tissue, more experiments are still needed to evaluate the therapeutic effect of the FePt@MMT-MIT system for the transferred cells in the future.

## Conclusion

This research focused on CTAB used to modify MMT, and then FePt@MMT nanocomposites with different magnetic properties were synthesized using a one-pot method, which changed the MMT ratio. Through the analysis, the crystal structure, microstructure, surface properties, magnetic properties, biocompatibility, adsorption properties, electrochemical properties, magnetocaloric effect, and magnetic resonance imaging were analyzed. Based on the data obtained in this study, the following conclusions, including: First, the one-pot method makes the prepared FePt@MMT with the advantages of low toxicity and high boiling point of triethylene glycol. Second, the adjustment of the MMT ratio was sufficient to increase the saturation magnetization of composite nanomaterials, which benefit magnetic hyperthermia and related applications of MRI imaging. Third, nanocomposite, the FePt@MMT's adsorption of MIT enables it to enhance the contrast of T2-weighted MRI using magnetic separation. Finally, welled magnetic and adsorption properties support the FePt@MMT-MIT nanocomposite, its potential effects for related treatment in biomedical applications.

Syntheses, characterization, and STEM recording data of all new compounds and experimental section details. Moreover, further in vitro and in vivo results are also presenting in Additional file 1.

## Supplementary Information

**Additional file 1.** Additional figures and tables.

**Additional file 2.** Additional STEM file.

## Data Availability

The data generated or analyzed during this study are included in the manuscript and additional information files.
