# Supplementary material for: Magnetically guided theranostics: montmorillonite-based iron/platinum nanoparticles for enhancing in situ MRI contrast and hepatocellular carcinoma treatment
Source: J Nanobiotechnology. 2021 Oct 9;19:308. doi: 10.1186/s12951-021-01052-7 (PMC8501633; doi:10.1186/s12951-021-01052-7)
Supplement: Supplementary file 1 — Additional file 1. Additional figures and tables. [file 12951_2021_1052_MOESM1_ESM.docx]

**Supporting Information**

**Magnetically Guided Theranostics: Montmorillonite-Based Iron/Platinum Nanoparticles for Enhancing *in Situ* MRI Contrast and Hepatocellular Carcinoma Treatment**

Ming-Hsien Chan,^‡^ Chih-Ning Lu,^‡^ Yi-Lung Chung, Yu-Chan Chang, Chien-Hsiu Li, Chi-Long Chen*, Da-Hua Wei*, and Michael Hsiao*

Michael Hsiao, Ming-Hsien Chan, and Chien-Hsiu Li − *Genomics Research Center, Academia Sinica, Taipei 115, Taiwan*

Chih-Ning Lu − *Department of Chemistry,* *Saint Michael's College, Vermont 05439, USA*

Da-Hua Wei and Yi-Lung Chung − *Graduate Institute of Manufacturing Technology and Department of Mechanical Engineering, National Taipei University of Technology, National Taipei University of Technology, Taipei 106, Taiwan*

Yu-Chan Chang *− Department of Biomedical Imaging and Radiological Sciences, National Yang Ming Chiao Tung University, Taipei 112, Taiwan*

Chi-Long Chen − *Department of Pathology, College of Medicine and Department of Pathology, Taipei Medical University Hospital, Taipei 110, Taiwan*

Michael Hsiao − *Department of Biochemistry, College of Medicine, Kaohsiung Medical University, Kaohsiung 807, Taiwan*

*Corresponding Authors:

Michael Hsiao, ORCID: orcid.org/0000-0002-6131-5706; Email: [mhsiao@gate.sinica.edu.tw](mailto:mhsiao@gate.sinica.edu.tw)

Da-Hua Wei, ORCID: orcid.org/0000-0001-7603-3739; Email: : [dhwei@ntut.edu.tw](mailto:dhwei@ntut.edu.tw)

Chi-Long Chen, ORCID: orcid.org/0000-0003-2875-1669; Email: : [chencl@tmu.edu.tw](mailto:chencl@tmu.edu.tw)

**Supporting Figure 1**


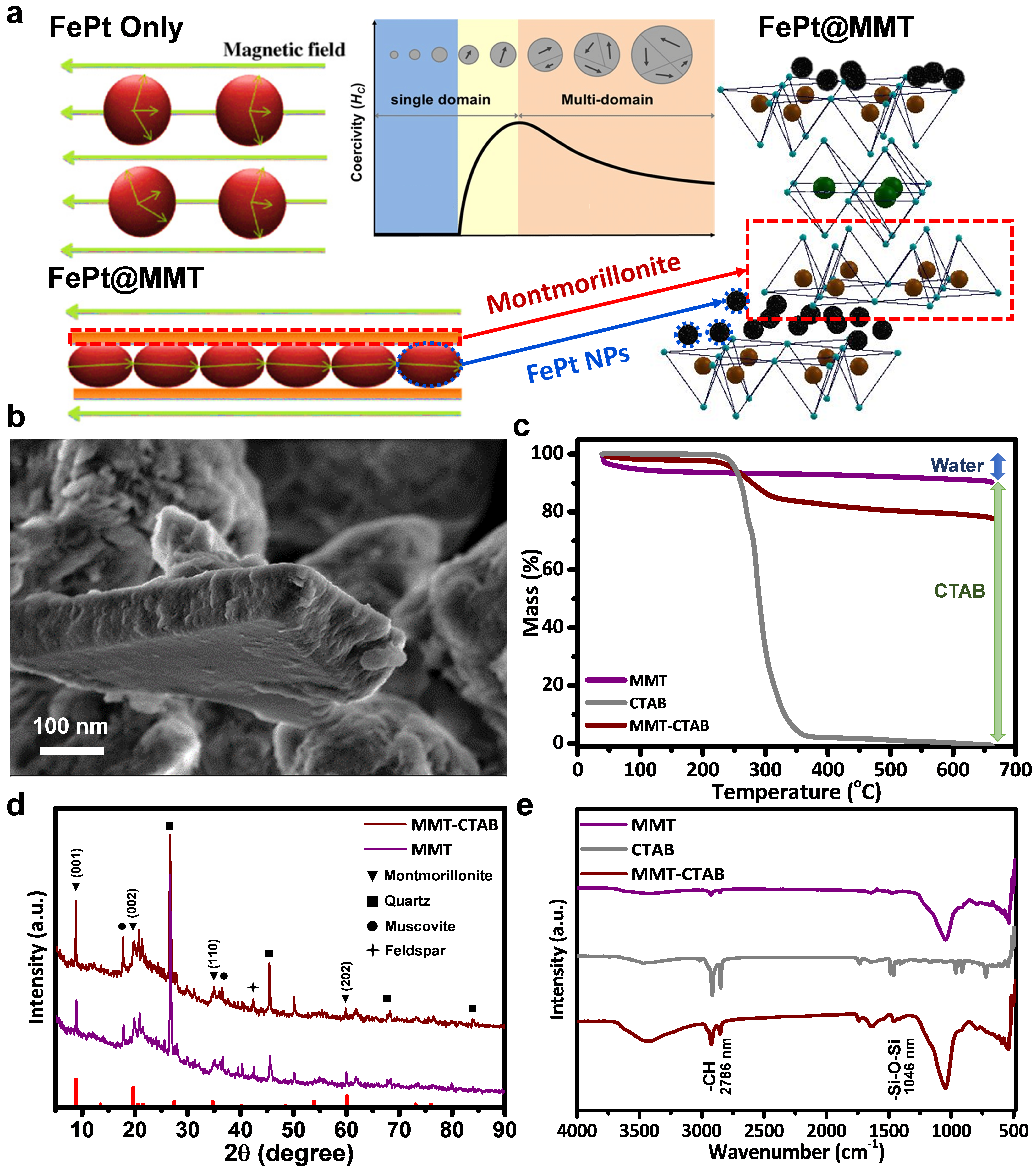


**Figure S1. The characterization of MMT and CTAB-modified MMT.** (a) The schematic shows the possible mechanism of layer-shaped MMT limiting the space FePt NPs and increasing the magnetic properties. (b) SEM image of CTAB-modified MMT. (c) TGA analysis evaluates the weight loss after removing the CTAB molecules. (d) The XRD patterns of MMT and CTAB-modified MMT. (e) FTIR spectrum detection of the Si–O and C–H functional groups have been successfully modified with MMT.

**Supporting Figure 2**


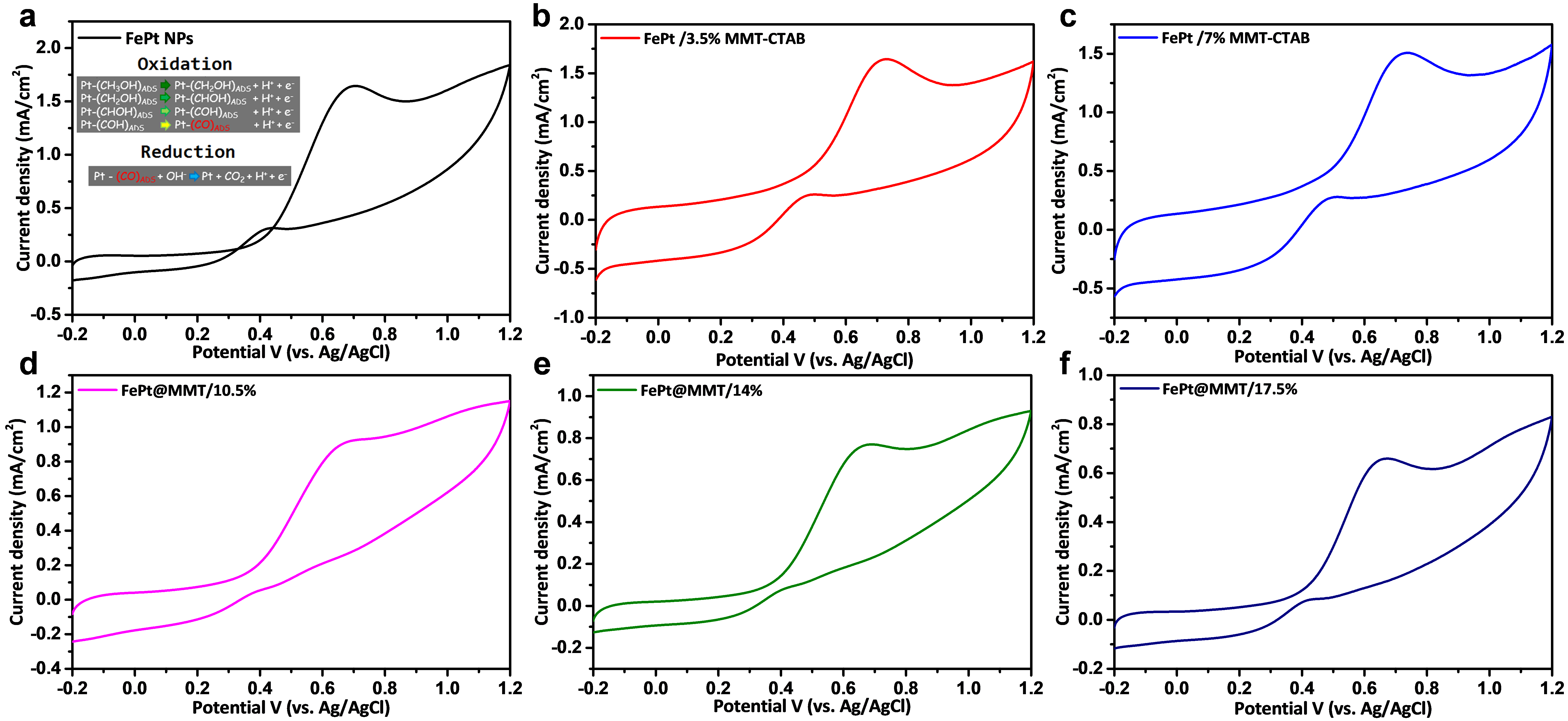


**Figure S2. Experimental results of electrochemical detection.** Cyclic voltammetry of series (a) FePt NPs, (b) FePt@MMT/3.5%, (c) FePt@MMT/7%, (d) FePt@MMT/10.5%, (e) FePt@MMT/14%, and (f) FePt@MMT/17.5% nanocomposites analysis (Insert: Direct methanol fuel cell reaction).

**Supporting Figure 3**


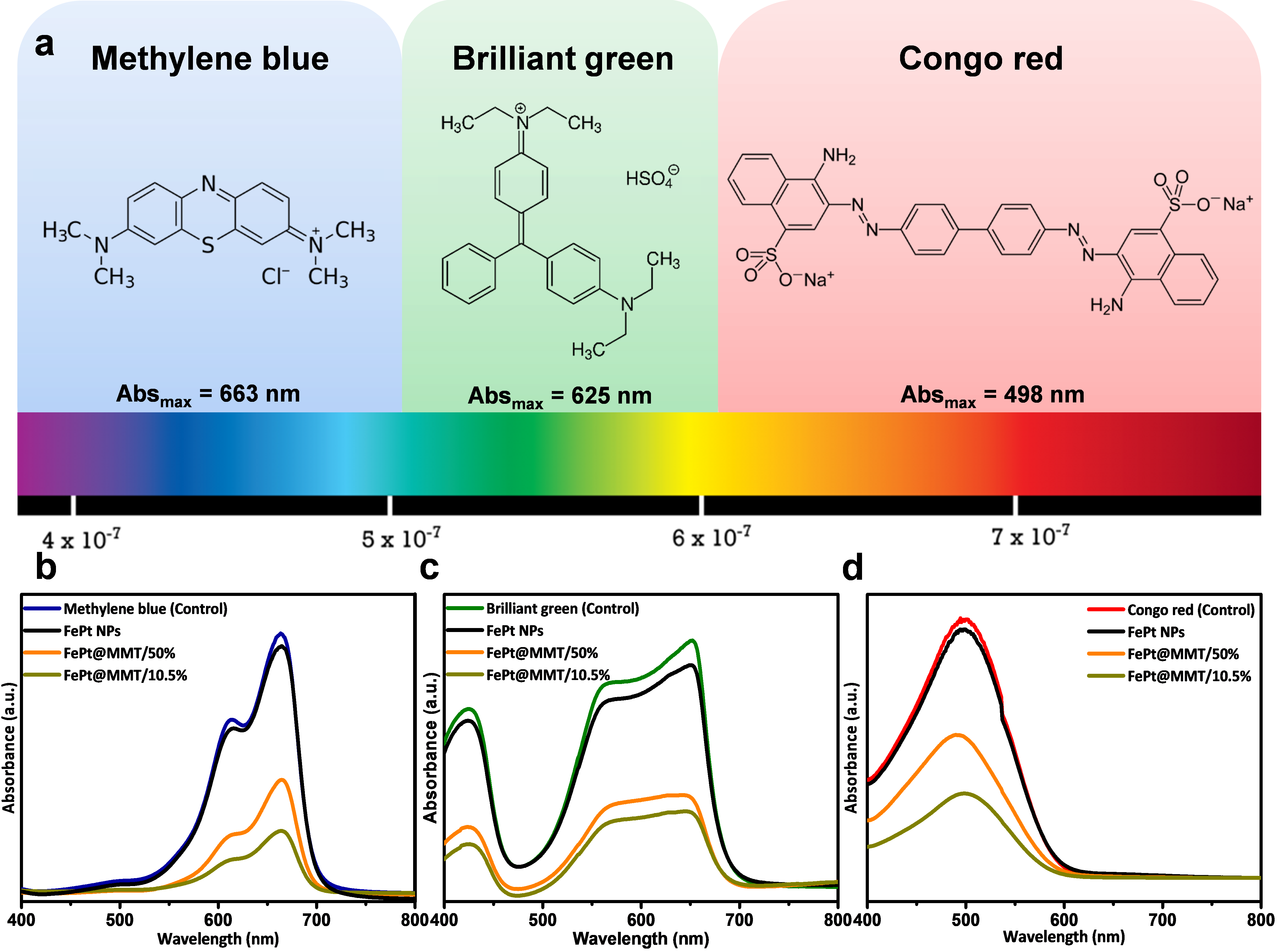


**Figure S3. Experimental results of dye adsorption with FePt@MMT.** The change of (a, b) methylene blue, (a, c) brilliant green, and (a, d) Congo red concentration after adding FePt@MMT and CTAB-modified FePt@MMT nanocomposites, was measured ultraviolet-visible absorption spectrometer to judge the ability of the sample to adsorb dyes.

**Supporting Figure 4**


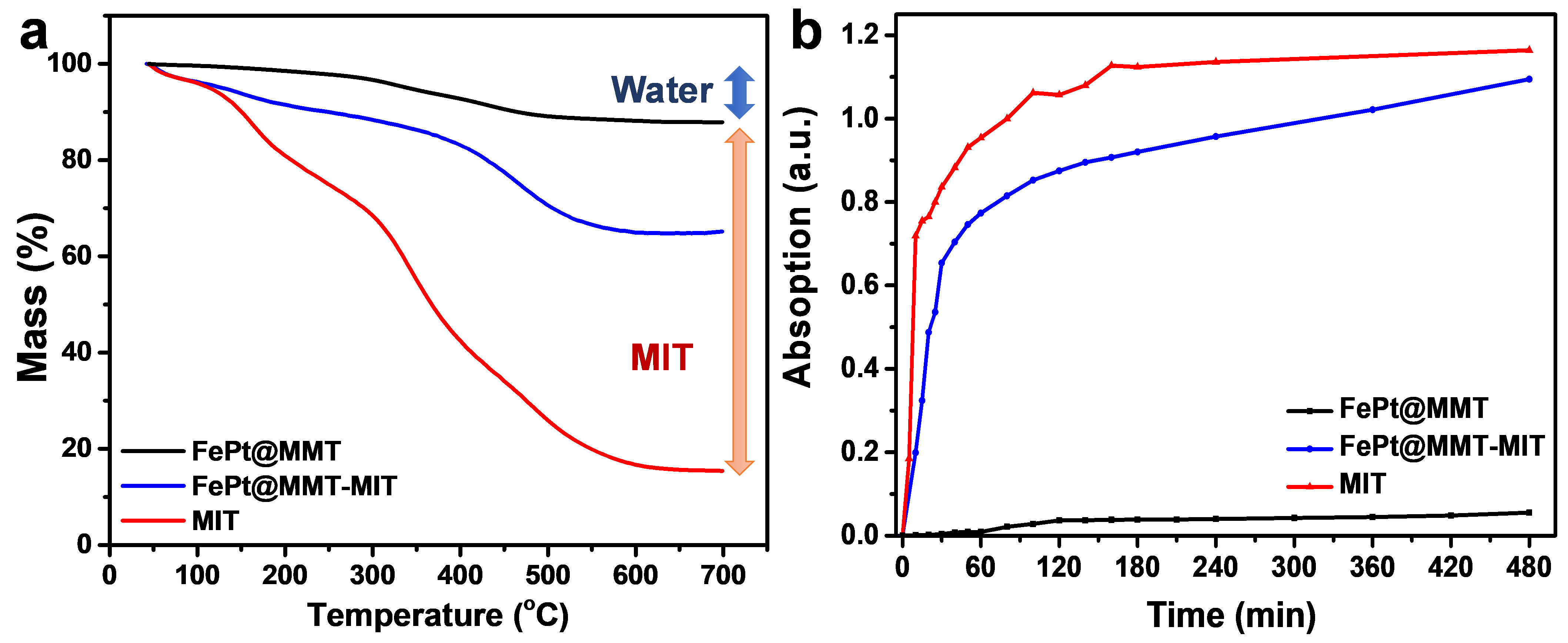


**Figure S4. Loading amount and release rate of MIT drugs.** (a) TGA data analyzed the drug-carrying capacity to analyze the loading ratio of each sample to the drug MMT. (b) The drug release was measured by UV-Vis spectroscopy to determine the absorption intensity of the MMT drug at each time point at 650 nm.

**Supporting Figure 5**


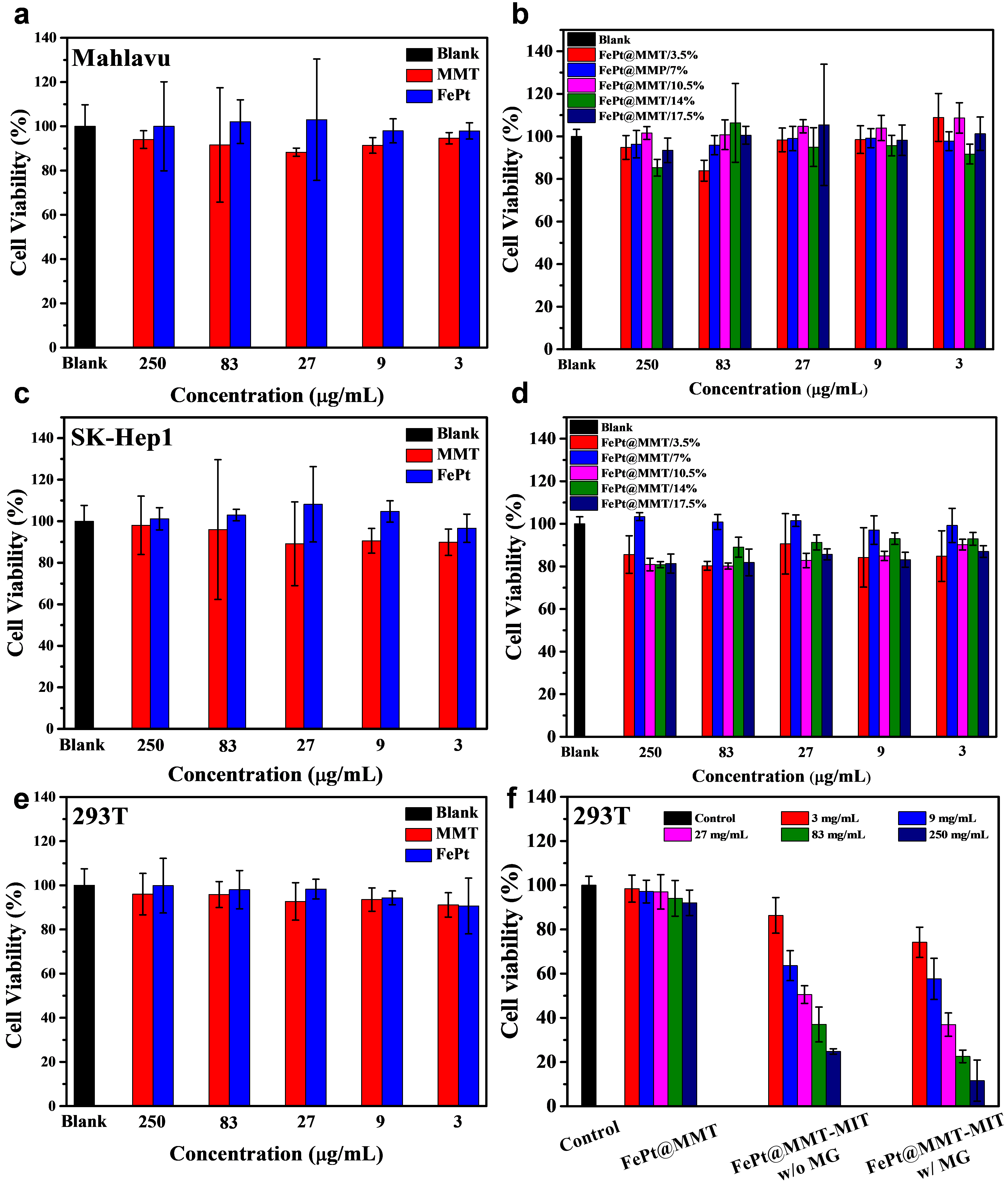


**Figure S5. Cell viability analysis.** The toxicity of FePt NPs and FePt@MMT nanocomposites were tested and stored at different concentrations for 48 hours. The results showed that the effect of different concentrations on cell viability was not significantly reduced, and the cell survival rate was maintained above 80%. Prove that FePt NPs and FePt@MMT nanocomposites have good biocompatibility in (a, b) Mahlavu, (c, d) SK-Hep1, and (e) 293T. Moreover, the different treatment groups were evaluated the (f) cell toxicity of 293T.

**Supporting Figure 6**

**Figure S6. Long-term IVIS results of orthotopic implant-Mahlavu cells for 6 weeks’ evaluation.** (a) A total of four groups of mice were Control, MIT, FePt@MMT, and FePt@MMT-MIT for IVIS long-term tracking with the Mahlavu HCC cell line. Orthotopic SK-Hep1 cell line implant and evaluate with the (b) IVIS result and photo images of liver tissues. (c) The H&E staining of orthotopic SK-Hep1 liver tissues.

**Supporting Table 1**

**Table S1.** Ms, ΔT/Δt and SAR values of FePt NPs and FePt@MMT nanocomposites.

| **Sample** | **m_Sample_**  **(g)** | **m_water_**  **(L)** | **M_s_**  **(emu/g)** | **ΔT/Δt**  **(℃/s)** | **SAR**  **(W/g)** |
| --- | --- | --- | --- | --- | --- |
| **FePt NPs** | **6×10^-3^** | **1×10^-3^** | **14.67** | **0.0193** | **13.37** |
| **FePt@MMT/3.5%** | **6×10^-3^** | **1×10^-3^** | **17.21** | **0.0303** | **21.02** |
| **FePt@MMT/7%** | **6×10^-3^** | **1×10^-3^** | **19.69** | **0.0341** | **23.64** |
| **FePt@MMT/10.5%** | **6×10^-3^** | **1×10^-3^** | **24.54** | **0.0574** | **39.74** |
| **FePt@MMT/14%** | **6×10^-3^** | **1×10^-3^** | **18.37** | **0.0319** | **22.08** |
| **FePt@MMT/17.5%** | **6×10^-3^** | **1×10^-3^** | **15.41** | **0.0250** | **17.33** |

**Supporting Table 2**

**Table S2.** The change UV-Vis value of solution concentration with different dyes after adsorption of nanoparticles for 10 min.

| **Sample**  **C/C_0_** | **Control** | **FePt NPs** | **FePt@MMT 50%** | **FePt@MMT 10.5%** |
| --- | --- | --- | --- | --- |
| **Concentration of Methylene blue (%)** | **100** | **95.03** | **45.58** | **23.93** |
| **Concentration of Brilliant green (%)** | **100** | **90.31** | **37.86** | **32.31** |
| **Concentration of Congo red (%)** | **100** | **96.16** | **54.28** | **32.65** |
